# Scanning Bessel beam microscopy with a protected and corrective objective for solvent-cleared large samples

**DOI:** 10.1016/j.isci.2026.116358

**Published:** 2026-06-12

**Authors:** Chia-Ming Lee, Po-Yen Lin, Yu-Ting Tseng, Xuejiao Tian, Chiao-Hui Tu, José Jiun-Shian Wu, Hsin Chen, Yi-Fen Cheng, Po-Ting Lin, Tung-Han Hsieh, Tzyy-Nan Huang, Tsan-Ting Hsu, Yijuang Chern, Yi-Ping Hsueh, Bi-Chang Chen

**Affiliations:** 1 Research Center for Applied Sciences, Academia Sinica, Taipei 11529, Taiwan; 2 Institute of Cellular and Organismic Biology, Academia Sinica, Taipei 11529, Taiwan; 3 Institute of Biomedical Sciences, Academia Sinica, Taipei 11529, Taiwan; 4 Institute of Molecular Biology, Academia Sinica, Taipei 11529, Taiwan

**Keywords:** imaging equipment, chemistry, applied sciences

## Abstract

Emerging imaging strategies, such as tissue clearing and advanced light-sheet modalities, are transforming the visualization of cell differentiation, tissue reorganization, and morphogenesis in both model and non-model organisms, thereby driving future discoveries of conserved developmental programs and species-specific innovations. In this study, we present a versatile Bessel light sheet microscopy (BLX) system compatible with both immersion and capped air objectives, optimized for whole-mount three-dimensional imaging of cleared tissues. We demonstrate that autofluorescence-based light-sheet imaging enables reliable visualization of CUBIC-R-cleared mouse embryos, revealing microanatomical features such as nephrons during developmental stages. To support solvent-based clearing protocols, we designed a protective lens cap that shields objectives from solvent erosion and economically converts standard air objectives into high-performance immersion objectives. Using this capped BLX configuration, we achieved cellular-resolution imaging of whole PEGASOS-cleared mouse brains. The system’s simplified design and open-source availability promote broader adoption for diverse applications in developmental biology.

## Highlights

• An economical Bessel light-sheet system protecting air objectives from solvent erosion
• Reliable anatomical visualization via autofluorescence in model and non-model organisms
• Capability to resolve fine developmental features like individual nephrons
• Neuronal activity mapping in whole PEGASOS-cleared brains via capped BLX microscopy

## Graphical abstract

**Figure:**
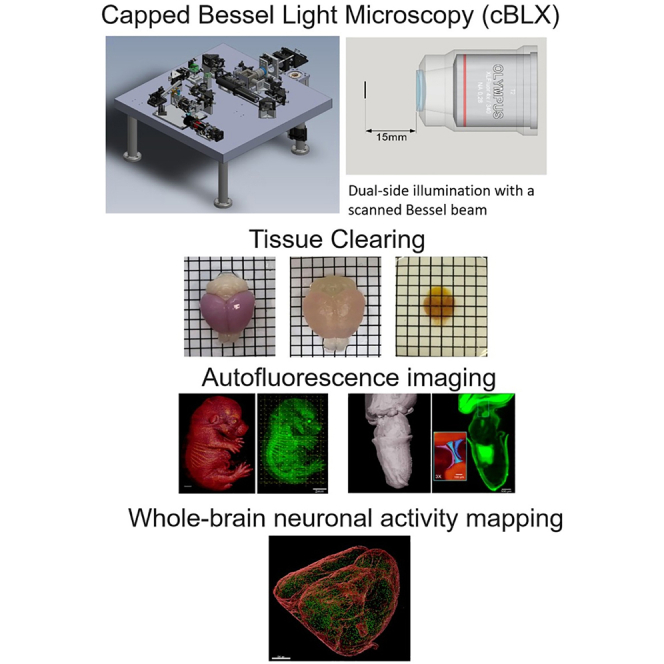

## Introduction

The visualization of cell differentiation, tissue reorganization, and morphogenesis is indispensable for advancing developmental biology, both in canonical model organisms that provide well-established genetic and experimental frameworks, and in non-model organisms that uncover lineage-specific innovations and broaden evolutionary perspectives.^1^ These dynamic processes are often distributed over broad spatial and temporal scales, posing cross-scale challenges for traditional microscopy techniques. With the advent of emerging imaging and sample preparation technologies, the intricate events underlying embryonic development have been increasingly revealed with unprecedented detail.^2^^,^^3^^,^^4^^,^^5^ However, as embryogenesis progresses, specimens often become opaque due to the accumulation of pigments, the development of hard tissues, and the formation of components with heterogeneous refractive indices. Endogenous pigments such as hemosiderin, melanin, and lipofuscin obstruct light transmission, while mineralized structures like bone and dental tissues further impede optical access. Additionally, variations in the refractive index among tissue components lead to light scattering, which limits imaging depth. Non-model organisms such as cephalopods, which is the origin of neuroscience and gained more attention recently, still lack specific and comprehensive biomarkers to well visualize the details for better evaluation and comparison during the developmental process.^6^^,^^7^

To overcome these challenges, tissue-clearing techniques have been developed to render specimens optically transparent, thereby enabling the application of three-dimensional (3D) imaging methods for visualizing tissue architecture in intact samples.^8^^,^^9^^,^^10^^,^^11^^,^^12^ For cleared samples, conventional fluorescence imaging techniques such as widefield (WF) and confocal microscopy are commonly employed. However, optimizing image quality in these systems often requires trade-offs between spatial resolution, photodamage, and imaging speed. For instance, WF microscopy allows rapid image acquisition through camera-based detection but suffers from poor axial resolution, limiting its ability to resolve 3D features. In contrast, confocal microscopy provides improved spatial resolution and optical sectioning but is inherently slower due to point-scanning acquisition.

Light sheet fluorescence microscopy (LSFM) has emerged as a powerful alternative for 3D imaging, offering high spatial and temporal resolution.^13^ It has been successfully applied to diverse biological contexts, including neural activity mapping,^14^ embryonic development,^15^^,^^16^ cell biology,^17^^,^^18^ and pathology.^19^ In traditional LSFM, illumination and detection are decoupled by using two orthogonally arranged objectives. A thin sheet of light is typically generated using either a cylindrical lens^16^ or a rapidly scanned Gaussian beam,^20^ projected into the sample via the illumination objective. Fluorescence from the illuminated plane is collected by the perpendicular detection objective and recorded by a camera. This configuration provides optical sectioning and fast acquisition, making LSFM ideal for dynamic 3D imaging.

Despite its advantages, conventional LSFM faces challenges when imaging large volumes at high resolution. The field of view (FOV) is limited by the axial extent of the light sheet, which is inversely related to its thickness.^21^^,^^22^^,^^23^ To achieve a larger FOV, Gaussian light sheets typically employ low numerical aperture (NA) illumination objectives, which result in thicker light sheets and reduced optical sectioning. Techniques such as focus-tunable lenses^24^ and remote focusing^25^ can extend the illumination focus along the propagation axis, but require scanning during camera exposure, leading to reduced pixel dwell time. Consequently, higher laser power or longer exposures are often necessary to achieve adequate signal intensity, increasing the risk of photodamage.^26^

Recently, several LSFM platforms have been developed to provide large FOV with diffraction-limited resolution. For example, a recent extension using a curved light sheet has achieved a centimeter-scale FOV with 1.0 μm resolution.^27^ However, it needs a costumed design objective lens and relative slow image acquisition due to line detection. Axially scanned light sheet was developed to improve the spatial resolution of the conventional LSM with isotropic 3D spatial resolution.^28^ However, it requires precise synchronization between the axial scanning device and the rolling shutter of detection scientific Complementary Metal-Oxide-Semiconductor (sCMOS) camera. These platforms are sophisticated and expensive, which limits their adoption by end users.

Self-reconstructing light beams, such as Bessel and airy beams,^29^^,^^30^^,^^31^ offer a compelling solution due to their propagation-invariant intensity profiles. A Bessel beam is generated by projecting an annular pattern onto the back focal plane of the objective, resulting in a needle-shaped focus along the propagation axis. Scanning this focus laterally creates a uniformly thin light sheet across a large FOV. Moreover, Bessel beams maintain their structure over long distances and can self-reconstruct after encountering obstructions, reducing striping artifacts and enabling deeper imaging in scattering tissues.^32^^,^^33^

In this study, we integrated tissue-clearing techniques with Bessel light sheet microscopy (BLX) to create a versatile and easy-to-build imaging platform for high quality 3D visualization of large biological samples. We designed a protective lens cap to shield the objective lens from solvent damage while simultaneously correcting for optical aberrations. Compared with commercial immersion objectives, our lens cap provides a cost-effective and long working-distance alternative that enables high quality imaging. Using this system, we successfully imaged clear, unobstructed brain imaging cocktails (CUBIC-) and polyethylene glycol (PEG)-associated solvent system (PEGASOS-) cleared samples, including entire adult mouse brains and embryos, revealing detailed 3D reconstructions of organs such as the brain, lungs, and kidneys.

## Results

### Design of the imaging system

Inspired by recent open-access light sheet microscopy platforms,^34^^,^^35^^,^^36^^,^^37^^,^^38^ we developed a simplified BLX system designed for accessible, high-throughput 3D imaging in developmental biology.

**Figure 1:**
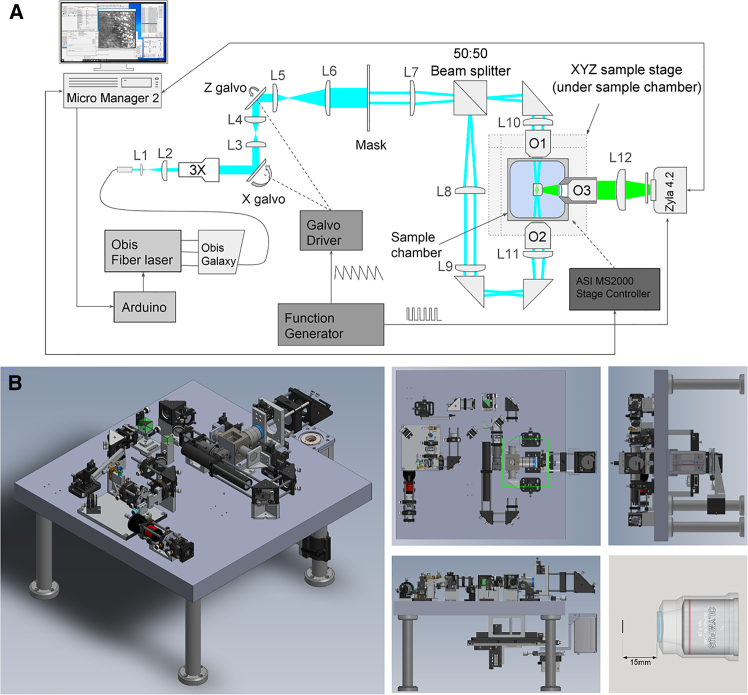
Design of the capped Bessel light sheet microscope (A) Schematic diagram of the cBLX system, featuring dual-side Bessel beam illumination and orthogonal fluorescence detection. A 488 nm laser is expanded and shaped into an annular profile using a beam expander, galvo mirrors, and a ring mask. The beam is split and directed through two excitation objectives to form Bessel light sheets from both sides of the sample. fluorescence emission is collected by a perpendicular detection objective and projected onto an sCMOS camera. A custom-designed lens cap adapts a standard air objective for solvent-based imaging, extending working distance and correcting aberrations. (B) CAD model of the assembled cBLX system, showing the optical layout and custom imaging chamber. Major components include the laser module, scanning mirrors, relay lenses, dual-side excitation paths, detection optics, and motorized sample stage.

The system minimizes the need for complex custom hardware and software while retaining essential light sheet functions. As illustrated in Figure 1 and Video S1, the microscope is operated through Micro-Manager, an open-source software package with broad support for commercial microscope components. Signal control specific to light sheet operation—such as galvanometer scanning, and synchronization with image acquisition—is handled by a programmable function generator (RIGOL DG1022Z), which delivers synchronized waveforms to both the galvo mirrors and the camera. This ensures tight timing between illumination and detection cycles. A complete list of components, optical elements, and control electronics is provided in the supplemental information.

Video S1. 3D animation of cBLX, related to Figure 1

Excitation was provided by a continuous-wave 488 nm laser. The laser beam was initially expanded to a 1/e^2^ diameter of approximately 6 mm using a two-lens beam expander (L1 = 8 mm, L2 = 25 mm), followed by a 3× Galilean beam expander (Thorlabs, GBE03-A). The expanded beam was directed to a scanning unit consisting of a pair of galvanometer mirrors, optically conjugated via relay lenses (L3 = 25 mm, L4 = 25 mm). To shape the beam into a Bessel-like profile, the scanned beam was relayed through an optical mask, positioned conjugate to both galvo mirrors via additional relay lenses (L5 = 60 mm, L6 = 80 mm). The resulting ring-shaped beam was split into two paths using a 50:50 non-polarizing beamsplitter, enabling dual-side illumination. Each beam was directed to the back focal plane of an excitation objective via a set of relay lenses (L7-L11, f = 100 mm) to form a symmetric Bessel light sheet.

To address common imaging artifacts, such as stripe patterns caused by light scattering and absorption heterogeneity in cleared tissues,^39^ we implemented dual-side illumination in combination with a scanned Bessel beam. This strategy increases the angular diversity of the excitation path, enabling more uniform and omnidirectional illumination. As a result, stripe artifacts are substantially suppressed, and image quality is enhanced.^40^^,^^41^ The use of a scanning Bessel beam is inherently accompanied by side lobes effect, which can lead to increased out-of-focus excitation. Increasing the beam ring thickness reduces side lobe intensity. However, this comes at the expense of a shortened axial extent of the Bessel beam, thereby limiting the usable FOV.^17^ In scanning Bessel light sheet system, this effect represents a trade-off between FOV and optical sectioning capability. To mitigate side lobe effect, we optimized the beam ring dimensions to achieve a balanced compromise between these two parameters. Furthermore, our dual-side illumination configuration helps compensate for the reduced Bessel beam length and associated loss in FOV.

The sample was immersed in a custom-designed imaging chamber filled with an imaging buffer and mounted on a long-travel-range motorized stage (ASI MS2000). Fluorescence signals are collected through a detection objective (Nikon 20× /1.0 Glyc), passed through a tube lens (f = 150 mm for 20× obj.), and captured by Andor Zyla 4.2 PLUS sCMOS camera, depending on the experimental configuration. For large-field imaging, especially when surveying entire embryos or adult mouse organs, a 4× Olympus objective (tube lens f = 300 mm) equipped with a custom-designed lens cap is employed. This cap not only protects the objective from solvent exposure during tissue clearing but also corrects for optical aberrations, enabling high-quality imaging across a significantly expanded FOV. This dual-mode configuration allows rapid switching between high-magnification cellular imaging and low-magnification overview scans.

### Tissue clearing combined with BLX enables whole-organism imaging

**Figure 2:**
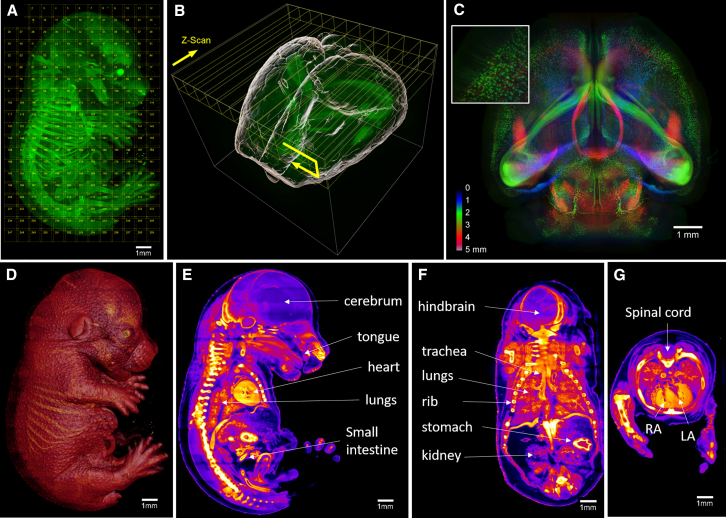
High-resolution autofluorescence imaging of CUBIC-R-cleared E15.5 mouse embryos and Thy1-YFP-H mouse brains (A) Maximum intensity projection of a whole CUBIC-R-cleared E15.5 mouse embryo. (B) Surface rendering of a Thy1-YFP-H transgenic mouse brain. (C) Whole-brain image with depth-coded fluorescence intensity. A magnified inset shows the labeled neuron. (D) Surface rendering of the E15.5 mouse embryo. (E–G) Digital sections extracted from the 3D image along the sagittal (E), coronal (F), and transverse (G) planes. Scale bars: 1 mm.

Using our cBLX system in combination with both the CUBIC-R and PEGASOS tissue clearing protocols, we performed high-quality volumetric imaging of large mouse specimens including whole E15.5 embryos and adult brains. Large-scale 3D datasets were acquired through multi-tile scanning to overcome the limited FOV of the objective lens. To cover the entire sample, we defined XY boundaries and used Micro-Manager’s “create grid” function to generate an automated tile array. The sample was moved in the X and Y directions to acquire overlapping tiles, while z stack acquisition was achieved with a continuous vertical scan. The yellow grid in Figure 2B shows the tile positions, arranged in a snake-by-row pattern during acquisition.

**Figure 3:**
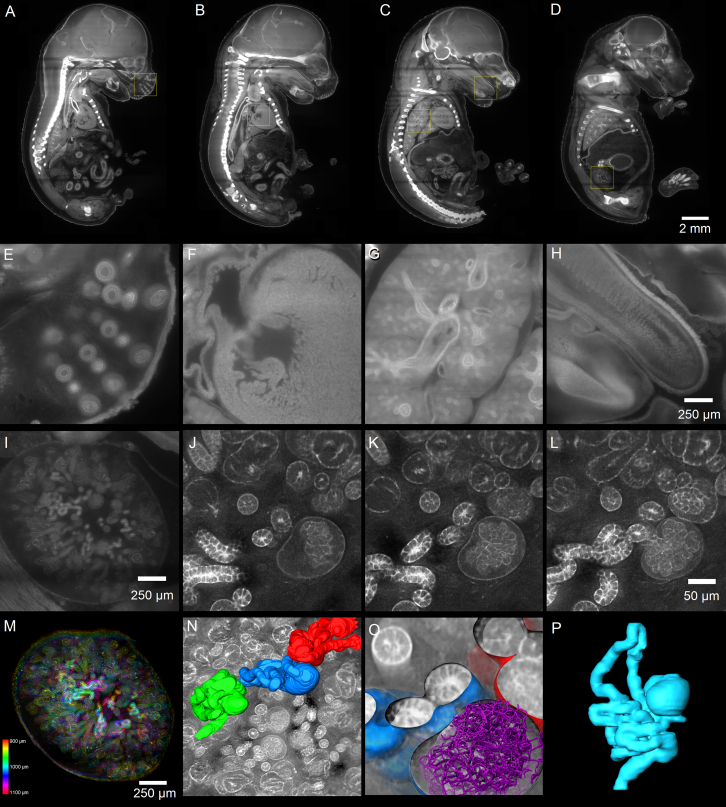
High-resolution light sheet autofluorescence imaging of a CUBIC-R-cleared E15.5 mouse embryo (A–D) Representative sagittal digital sections extracted from a reconstructed 3D image of the whole embryo. (E–I) High-resolution views of anatomical structures, including (E) vibrissal follicles, (F) heart, (G) lung, (H) tongue, and (I) kidney. (J–L) Visualization of early nephron development in the embryonic kidney. (M) Whole kidney autofluorescence image with color-coded depth information. (N) Image segmentation showing three individual nephrons. (O) Capillary walls (purple) inside Bowman’s capsule. (P) 3D rendering of segmented nephrons illustrating their spatial morphology.

Figure 2A demonstrates the full coverage of an E15.5 embryo cleared with CUBIC-R and imaged using autofluorescence, while Figures 2B and 2C shows an adult Thy1-yellow fluorescent protein (YFP)-H mouse brain cleared with PEGASOS. Figure 2B illustrates the acquisition layout, and Figure 2C presents a depth-coded rendering of the full processed volume. A complete embryo was reconstructed from 260 volumetric tiles, producing a final imaging volume of approximately 9 × 16 × 6 mm^3^, the detailed results are shown in Figure 3.

Embryonic day 15.5 (E15.5) marks a critical mid-gestational stage in mouse development, characterized by rapid tissue growth, organ differentiation, and structural maturation. At this stage, major organs—including the brain, lungs, liver, kidneys, and heart—are well-established, and the embryo reaches approximately 13–16 mm in crown-rump length. Neural development progresses rapidly, with the cerebral cortex undergoing active layering and expansion. Concurrently, the kidneys exhibit robust nephrogenesis, the lungs advance through the canalicular stage of branching morphogenesis, and ossification centers appear throughout the skeleton. This developmental richness makes E15.5 an ideal time point for whole-mount imaging to assess anatomical integrity and organogenesis.

Autofluorescence signals—originating from intrinsic biomolecules such as nicotinamide adenine dinucleotide + hydrogen (NADH), flavins, collagen, and elastin—allowed label-free visualization of major anatomical structures.^42^^,^^43^^,^^44^^,^^45^ As seen in Figures 2E–2G, digital cross-sections of the 3D volume reveal detailed internal features such as the brain, lungs, liver, and kidneys. This modality provides histological context and enables longitudinal or comparative developmental studies without the need for exogenous fluorescent labels. Moreover, the combination of tissue clearing and autofluorescence imaging in large cleared volumes offers an efficient and accessible approach to phenotype embryos, characterize organ development, and identify tissue-specific anomalies. Compared to conventional whole-body imaging methods like micro-computed tomography (micro-CT), BLX provides subcellular optical resolution, enabling analysis of finer structures including vascular networks and early nephron development.^46^

### High-resolution autofluorescence imaging of the developing kidney

Zooming in on the embryonic kidney, we leveraged autofluorescence imaging to investigate nephrogenesis in the E15.5 mouse embryo. This stage represents a critical window for kidney development, during which nephron progenitors are actively differentiating into mature nephrons. The total number of nephrons is established prenatally and remains fixed throughout life, making accurate developmental quantification crucial for understanding susceptibility to renal disease.

Using digitally extracted cross-sections and 3D surface renderings (Figure 3, Video S2), we visualized internal organ architecture including vibrissal follicles, heart, lungs, tongue, and kidneys (Figures 3E–3I). Within the kidney, we resolved the structure of developing nephrons, including renal corpuscles and nephron tubules (Figures 3I–3P). Figures 3N–3P highlights individual autofluorescent nephrons and capillary walls inside Bowman’s capsules. These structures are clearly distinguishable without immunostaining, underscoring the capability of BLX combined with tissue clearing to capture fine anatomical detail at organ-wide scales.

Video S2. 3D rendering of the CUBIC-R cleared whole E15.5 mice and its kidney, related to Figures 2 and 3

Autofluorescence imaging is particularly advantageous in cleared embryonic tissue, where antibody penetration for immunostaining is often inconsistent.^47^ The uniform signal allows for high resolution mapping of renal structures throughout the entire kidney. This supports downstream applications such as morphometric quantification of nephron number and size, which are key indicators of renal health and future disease risk.^48^

### Adult whole brain imaging with cBLX and PEGASOS clearing

To further validate the versatility of our capped Bessel light sheet microscopy (cBLX) system, we applied it to adult mouse brain imaging using the PEGASOS clearing protocol. PEGASOS is a powerful organic solvent-based clearing method that renders a wide variety of tissues optically transparent while preserving fluorescence signals (Figure S1). However, organic solvents are chemically aggressive and can damage objective lenses^49^; necessitating protective adaptations for optical components (Figure S2).

High-quality imaging of large cleared samples typically requires objectives with long working distances, high NAs, and large FOVs—a combination that is rare and often prohibitively expensive. To address this, we designed a custom curvature lens cap as shown in Figure S4 mounted on a 3D-printed holder that both shields the objective from solvent erosion and optically adapts a standard commercial air objective (Olympus, 4×, NA 0.28, dry) for immersion use. This cap provides a working distance of up to 15 mm. Compared to a flat window, the curvature of the cap reduces internal reflections and enhances light collection efficiency, resulting in improved image contrast, shown in Figures S3A and S3B. Notably, the signal-to-background ratio (SBR) improved significantly, increasing from approximately 2 to 7. This solution represents a low-cost, modular alternative to custom immersion objectives and enables high-quality imaging with extended working distances.

**Figure 4:**
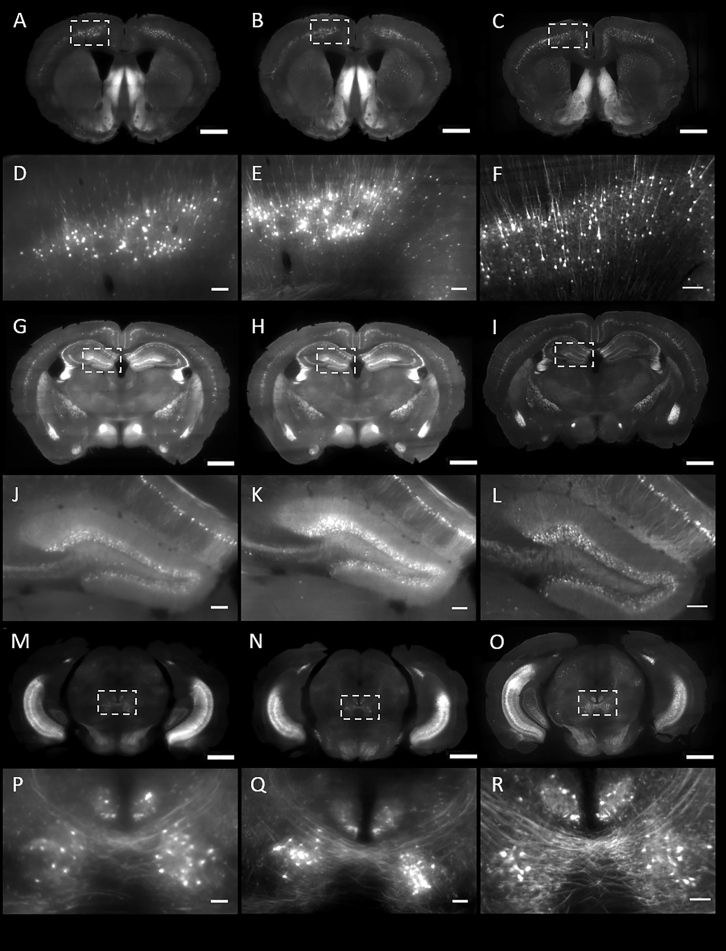
Comparison of Thy1-YFP mouse brain images acquired using different objective lenses (A, G, and M) Coronal optical sections of a PEGASOS-cleared Thy1-YFP mouse brain imaged using BLX equipped with a Nikon 20× immersion objective. (B, H, and N) Images acquired using a 4× dry objective with flat window interface. (C, I, and O) Images acquired using a 4× objective equipped with a custom-designed cap lens. (D–F) Zoom-in views of the regions indicated in panels (A–C), respectively. (J–L) Magnified views of selected areas in (G–I). (P–R) High-magnification regions extracted from (M–O). Scale bars:1 mm for (A–C), (G–I), and (M–O); 200 μm for (D–F), (J–L), and (P–R).

We evaluated the performance of this system using Thy1-YFP-H transgenic mice, which express YFP in a subset of neurons, primarily in the neocortex and hippocampus.^50^ The Thy1 promoter drives strong, cell-type-specific labeling, making it ideal for structural mapping. Using the 20× detection objective with 3.4 μm resolution and an exposure time of 85 ms, we successfully reconstructed the entire PEGASOS-cleared Thy1-YFP brain without requiring physical sectioning (Figures 4A, 4D, and 4G; Video S3). Stripe artifacts were notably absent throughout the imaging depth, and YFP-labeled somata and dendrites were clearly resolved in cortical layers (Figure 4B). While Thy1-YFP primarily labels layer 5 pyramidal neurons, recent studies have also reported labeling of additional interneuron subtypes.^51^

Video S3. 3D rendering of an entire PEGASOS-cleared (*n* = 1.52) thy1-YPF mice brain, related to Figure 4

Despite the clarity offered by PEGASOS, some image degradation occurred in deeper regions—particularly when imaging the hippocampus beneath the overlying cortex (Figure 4J). Similar results were observed when imaging using the 4× objective (Figure 4K) with 4.5 μm resolution and an exposure time of 85 ms via a flat window, highlighting persistent challenges in deep-tissue imaging due to residual scattering and attenuation.^45^

Nonetheless, the 4× objective with our designed lens cap enabled broad FOV imaging of the entire brain with the resolution of 3.5 μm while still resolving somata and dendritic structures (Figures 4C, 4I, and 4O). This configuration facilitated detailed mapping of dendritic networks and cortical projections while preserving imaging depth. Notably, when imaging with the 20× objective, 88 tiles were required to capture the full brain volume, generating over 2.5 TB of data. The acquisition process took 12 h, followed by additional time for stitching and registration. In contrast, the 4× configuration reduced the number of required tiles to 35 and completed the scan in approximately 4 h—achieving a 3-fold reduction in imaging time while maintaining sufficient resolution to identify neuronal morphology (Figures 4F, 4L, and 4R).

### Brain-wide functional imaging using activity-dependent c-Fos labeling

In addition to resolving neural circuit structure, mapping neuronal activity is essential for understanding how the brain responds to external stimuli and behavioral states. The immediate-early gene c-Fos is a well-established marker of neuronal activation, reflecting recent neural activity with high spatial and temporal specificity.^52^ To assess the capability of our cBLX system for whole-brain activity mapping, we imaged Fos2A-iCreER;Ai75 double transgenic mice (hereafter referred to as Trap2 mice), in which tdTomato expression is driven by c-Fos promoter activity in neuronal nuclei.

**Figure 5:**
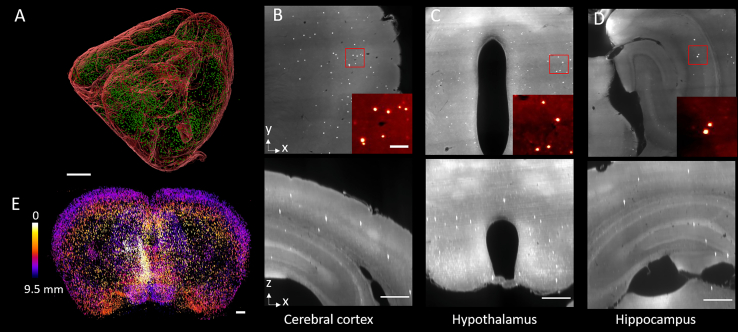
Whole-brain neuronal activity mapping in a PEGASOS-cleared Trap2 mouse brain using cBLX (A) 3D rendering of a PEGASOS-cleared mouse brain (excluding cerebellum and olfactory bulb), showing tdTomato-expressing neurons detected in Trap2 mice. Over 50,000 tdTomato-positive cells are visualized as green spots across the entire brain volume. (B–D) Representative images of tdTomato-labeled neuronal nuclei in the cerebral cortex (B), hypothalamus (C), and hippocampus (D), respectively. Insets measure 320 × 320 μm. Scale bar:100 μm. (E) Coronal view of a 3D c-Fos activity map, with *z* axis position color-coded according to the depth scale bars Scale bar: 500 μm for (A-E).

These Trap2 mice provide a powerful platform for identifying, monitoring, and manipulating neuronal ensembles engaged during defined behaviors or experimental conditions.^53^^,^^54^ Following behavioral activation and PEGASOS tissue clearing, we imaged the entire brain using cBLX to visualize tdTomato-labeled neurons at cellular resolution (Figure 5A). The resulting 3D rendering reveals widespread expression of tdTomato-positive nuclei throughout the brain. In total, over 50,000 c-Fos-activated neurons were detected across the imaged brain volume.

Each fluorescent cell was spatially mapped to specific anatomical brain regions by co-registering the image volume to the Allen Mouse Brain Atlas,^55^ enabling region-wise quantification of neuronal activation. Representative images of activated neurons in the cerebral cortex, hypothalamus, and hippocampus are shown in Figures 5B–5D. These data demonstrate that cBLX combined with PEGASOS clearing enables robust visualization of gene expression-based neuronal activation across diverse brain regions.

While c-Fos imaging is a powerful tool, it presents challenges due to high background autofluorescence and low expression signal in some cell populations. Traditional segmentation based on intensity thresholds often fails to distinguish weakly labeled nuclei from background noise. To address this, we implemented a deep learning-based segmentation algorithm (Cellpose),^56^ which allowed for accurate and automated detection of tdTomato-labeled cells across serial optical sections. This significantly improved our ability to quantify activity patterns reliably across the brain.

Overall, our results establish the potential of cBLX in conjunction with PEGASOS clearing for whole-brain functional mapping with cellular resolution. This platform provides a powerful approach for studying brain-wide activity, enabling correlations between structure, activity, and behavior in intact tissue volumes.

### Tissue clearance of bigfin reef squid embryos reveals developmental progression across stages 24 and 27

To further demonstrate the versatility of our autofluorescence-based volumetric imaging platform, we applied tissue clearance (CUBIC-R) to bigfin reef squid (*Sepioteuthis lessoniana*, *S. lessoniana*) (Loliginidae) embryos at two key developmental stages: stage 24 and stage 27. Previous studies indicate that the intercapsular embryonic development can be divided into 30 stages. The organogenesis began from the invagination and the mantle was visible in *S. lessoniana*. These stages span critical periods of cephalopod morphogenesis, including neural tube expansion, optic lobe development, and organ differentiation.

**Figure 6:**
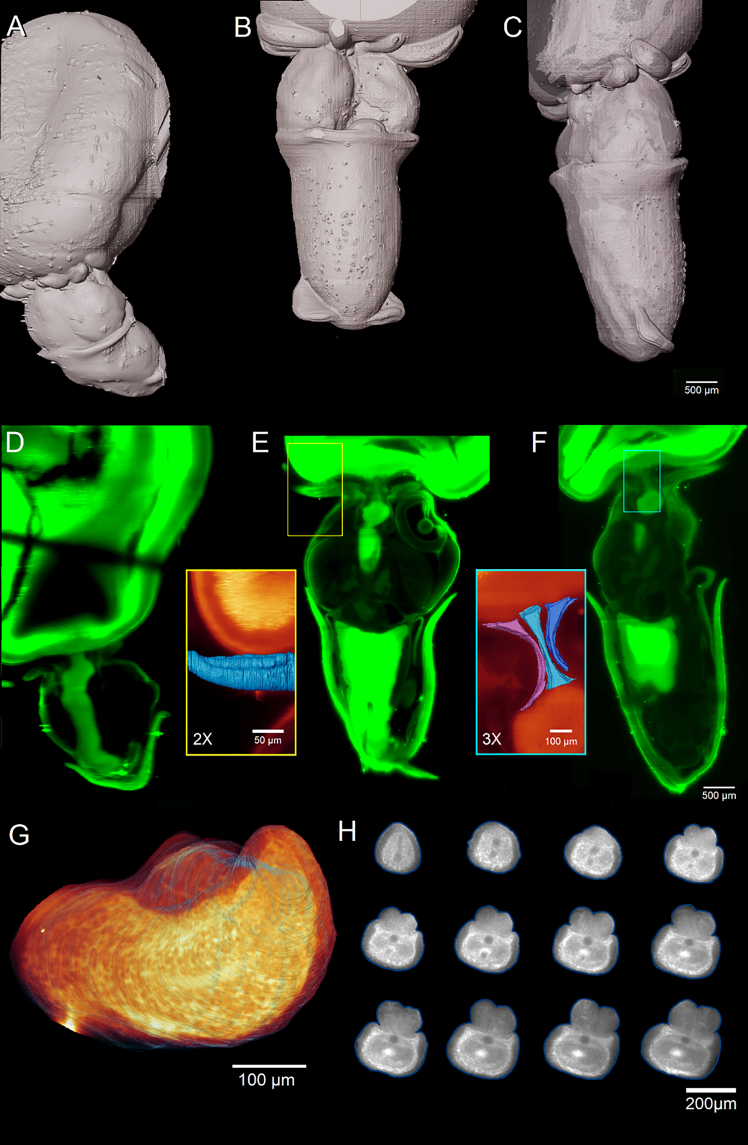
Autofluorescence imaging of squid embryos at developmental stages 24 and 27 Squid embryos at stage 24 and 27 were prepared using CUBIC-R tissue clearing and imaged with capped 4× objective. (A) Surface rendering of a stage 24 embryo generated via AMIRA, illustrating the structural details of the sample surface. (B and C) Dorsal and lateral views of stage 27 embryos, respectively. At this stage, the mantle and body are well-developed, with the yolk clearly connected to the head and mouth regions. (D and E) Autofluorescence section of the embryonic body, highlights the key anatomical features of above figures. The early eye and neural development at stage 24, optic lobe and arm primordia formation at stage 27. The inset is the segmentation reconstructed manually shown in 2×. The arm is manually segmented with a blue lines mask and shown in (G) and (H). (F) The autofluorescence image of the lateral view of the stage 27 squid embryos. The magnified inset highlights the structural linkage between the embryonic body and the yolk sac. Manual segmentation reveals that this connection consists of three major bundles originating from the yolk. The diameter each bundle is about 15 ± 5 μm, and total width of the three-bundle is about 110 μm. (G) Surface rendering of the stage 27 squid embryo arms, and (H) representative optical sections showing manual segmentations. The sucker bulb can be observed and segmented to reconstruct the structure. The arm diameter is from 204 ± 3 (upper left) to 306 ± 5 (lower right) μm, measured by the ImageJ.

Figure 6 shows representative coronal and horizontal sections of *S. lessoniana* clearance embryos acquired using autofluorescence detection on the cBLX system with capped 4× objective lenses. Autofluorescence originates from endogenous biomolecules such as elastin, chitin, and mitochondrial cofactors, which are naturally abundant in the tissue of the body and the mantle. This intrinsic signal enables high-contrast, label-free visualization of the invertebrate organism tissue architecture.

As shown in Figure 6A, at stage 24 (12–13 days after egg capsule deposition), the optic region was markedly swollen and the eye appeared pale yellow. The primordia of the ventral organs became prominent, and a distinct boundary was evident between the mantle, cephalic region, and yolk ball. The optic lobes, cephalic ganglia, and arm buds showed clear elaboration, with sucker primordia visible on the tentacles. The eyes were partially covered by a lid extending from the cephalic outer layer, indicating rapid regionalization of the nervous system. Internally, the ventral organs—including the gills, caecum, and hepatopancreas—were also discernible. Several ultrastructure such as the bulb of the sucker and the mouth-like linkage with the yolk can be observed with a clearing sample.

At stage 27 (16 days after egg capsule deposition) shown in Figures 6B and 6C, the chromatophores become intense and spread toward the tentacles. The eyes were fire-brick color. A significant ink feature became concentrated in the ink sac. An apparently established funnel tube and two types of chromatophores could be identified, as shown in Figures 6D and 6E. A distinctly red eye could be observed. It is worth noting that magnification microscopy highlights the connection between the yolk sac and the ovary. The autofluorescent connecting band represents the transport of nutrients shown in Figure 6F, and it can be manually segmented from the yolk sac into three main bundles, which is not observed and described previously.

Tissue clearing significantly enhanced resolution and reduced scattering, enabling deeper light sheet penetration and revealing cellular details within thick embryonic samples, as shown in Figures 6G and 6H. The preservation of structural integrity during clearance offers a powerful alternative to antibody-based staining, which is often limited by fewer worked antibodies and the poor penetration in marine invertebrates. Together, these data highlight the compatibility of tissue clearance with our light sheet system and underscore its potential in comparative developmental biology for both model and non-model marine organisms.

## Discussion

In this study, we demonstrate that LSFM, when combined with tissue-clearing techniques, enables volumetric imaging of large biological samples with cellular resolution. To address the optical and mechanical challenges associated with solvent-based clearing, we developed a custom-designed lens cap that protects objective lenses from organic solvent damage and corrects imaging aberrations. This approach effectively converts a commercial air objective into a high-performance immersion objective with a long working distance, significantly enhancing imaging depth and contrast in cleared tissues.

Our system’s versatility was demonstrated through multiple applications. We successfully imaged whole mouse embryos at embryonic day 15.5 (E15.5)—a mid-gestational stage marked by active organogenesis—using autofluorescence signals alone to reveal anatomical features such as the brain, lungs, liver, and kidneys. These results validate our platform for structural analysis of intact developmental specimens.

Beyond structural imaging, we also evaluated functional brain imaging using Trap2 transgenic mice, which express tdTomato in activated neurons via the c-Fos promoter. Following behavioral activation and PEGASOS clearing, we mapped over 50,000 tdTomato-positive cells across the entire brain with cellular resolution. Unlike c-Fos immunostaining, which captures all neurons expressing c-Fos protein at a specific time point, the TRAP2-mediated iCre recombination is strictly dependent on the presence of 4-OHT. Consequently, the resulting labeling is often more sparse and selective, representing highly activated ensembles rather than the entire c-Fos+ population. Region-specific activation patterns were co-registered to the Allen Brain Atlas, and deep learning-based segmentation allowed reliable quantification in high-background conditions. This demonstrates the system’s suitability for mapping activity-dependent gene expression in whole-brain volumes, which is critical for studying functional architecture and behaviorally relevant neural ensembles.

Importantly, we extended this platform to the bigfin reef squid (*S. lessoniana*), a non-model marine invertebrate system. Compared with widely used model organisms such as fruit fly (*Drosophila melanogaster*) or zebrafish (*Danio rerio*), non-model species often lack well-characterized molecular markers or require technically challenging and invasive labeling methods for long-term tracking of individual organelles. These limitations in both spatial resolution and temporal accessibility have historically restricted cephalopod developmental studies to conventional light microscopy approaches.^6^^,^^7^^,^^57^

Using tissue clearing (CUBIC-R), we imaged stage 24 and stage 27 bigfin reef squid embryos, resolving fine anatomical features such as arm buds, muscle fibers, yolk linkages, and optical elements without exogenous labeling.^58^^,^^59^ The clearing specimens, visualized via endogenous autofluorescence,^60^ preserved structural integrity and enabled high-resolution imaging of delicate tissues that are otherwise inaccessible due to light scattering and tissue density. These findings underscore the compatibility of tissue clearing with light sheet microscopy^61^ and establish this combined approach as a valuable tool for developmental anatomy in marine invertebrates and other non-model organisms.

Our cBLX system was deliberately engineered to be straightforward to construct and maintain, relying primarily on commercially available off-the-shelf components, open-source control software, and only minimal custom mechanical parts. This pragmatic, low-cost design strategy ensures that laboratories lacking extensive engineering expertise can readily replicate and adapt the setup for their own applications. The affordability and modularity of the cBLX platform make it particularly attractive for academic environments that seek to establish high-throughput, large-scale 3D imaging capabilities without investing in prohibitively expensive commercial solutions.^62^^,^^63^

Based on our comprehensive parts list, the total hardware cost of constructing a two-color laser cBLX system is approximately $35,545 USD. This estimate covers a scientific-grade sCMOS camera, a galvo scanner pair with drivers, a precision XYZ motorized stage, dual-wavelength (Diode-Pumped Solid-State) DPSS lasers for illumination, and all required optics, optomechanical mounts, and control electronics. Additional cost savings can be achieved by substituting lower-cost alternatives for select components, such as the camera or motion controller, depending on performance requirements and local resources. The simplified design allows for rapid setup, routine operation, and minimal maintenance, thereby lowering the technical barrier for adoption and supporting broad dissemination of advanced light-sheet imaging methods.

Together, these results establish the cBLX platform as a powerful, modular, and accessible solution for high quality structural and functional imaging across both model and non-model systems. The ability to seamlessly switch between high-magnification and large-FOV imaging modes, combined with compatibility with tissue clearing and expansion microscopy protocols, uniquely positions this system for integrated studies in developmental biology, neuroanatomy, and comparative embryology.^42^^,^^58^^,^^64^ Looking forward, coupling rich multimodal datasets with real-time neural-activity sensors will broaden applicability across diverse model systems and, critically, enable developmentally resolved, large-scale functional connectomics.

### Limitations of the study

The major limitation of the present approach is its incompatibility with deep tissue imaging in live specimens. One photon excitation is inherently limited by poor light penetration in 3D whole mount imaging, leading to degraded image quality. Although tissue clearing can circumvent this limitation, it requires fixed specimens for sample preparation, which precludes the observation of dynamic biological processes such as cell migration, lineage progression, and neuron activity events in real time. As such, the method is suited for capturing high-quality snapshot analysis rather than continuous or real-time functional imaging in deep tissue. However, this restriction could potentially be overcome by integrating multiphoton excitation with the cBLX system to enable deep-tissue imaging in live specimens. Nevertheless, the excessive power of the laser may prove damaging to living tissue. In addition, we demonstrate that autofluorescence imaging enables reliable visualization of CUBIC-R–cleared samples, revealing anatomical structures and their fine features. However, the non-specific nature of autofluorescence imaging makes it challenging to distinguish cell types and molecular identities. Moreover, signal intensity and spectral properties can vary across tissues, developmental stages, and species.

## Resource availability

### Lead contact

Requests for further information and resources should be directed to and will be fulfilled by the lead contact, Bi-Chang Chen (chenb01@as.edu.tw).

### Materials availability

This study did not generate new unique reagents.

### Data and code availability

• All data reported in this work will be shared by the lead contact upon request.
• The control macros are provided in supplementary supporting files.
• Other items including custom optical designs are provided in supplementary supporting files.

## Acknowledgments

The authors would like to acknowledge financial support from the 10.13039/100020595National Science and Technology Council of Taiwan (NSTC 113-2113-M-001-034 to B.-C.C.) and Academia Sinica of Taiwan (AS-TP-114-M01, AS-CFII-114-A12 to B.-C.C.).

## Author contributions

Conceptualization, P.-Y.L. and B.-C.C.; methodology, Y.-T.T., X.T., C.-H.T., J.J.-S.W., H.C., Y.-F.C., P.-T.L., T.-H.H., T.-N.H., T.-T.H., and B.-C.C.; investigation, C.-M.L. and X.T.; visualization, C.-M.L.; writing – original draft, P.-Y.L., and C.-M.L.; writing – review and editing, P.-Y.L., and B.-C.C.; funding acquisition, B.-C.C.; resources, Y.-P.H., Y.C., and B.-C.C.; supervision, P.-Y.L. and B.-C.C.

## Declaration of interests

The authors declare no competing interests.

## Declaration of generative AI and AI-assisted technologies in the writing process

During the preparation of this work, the authors used ChatGPT in order to improve the clarity and language of the manuscript. After using this tool, the authors reviewed and edited the content as needed and take full responsibility for the content of the final publication.

## STAR★Methods

### Key resources table

**Table:** 

REAGENT or RESOURCE	SOURCE	IDENTIFIER
**Experimental Model**		

Thy1-YFP-H transgenic mice	The Jackson Laboratory	No. 003782
Fos2A-iCreER knockin mice	The Jackson Laboratory	No. 030323
Ai75 reporter mice	The Jackson Laboratory	No. 025106

**Chemicals, peptides, and recombinant proteins**		

N,N,N,N-Tetrakis(2-Hydroxypropyl)ethylenediamine 98% (Quadrol)	Sigma-Aldrich	AL-122262-1L
Paraformaldehyde	Sigma-Aldrich	16005-1 KG
Ammonium hydroxide 25%	Sigma-Aldrich	Cat.no. 105432
tert-Butanol	Sigma-Aldrich	360538
Benzyl benzoate	Sigma-Aldrich	W213802
PEGMMA	Sigma-Aldrich	447943
CUBIC-R+ (for animals)	Tokyo Chemical Industry	Cat#T3740
CUBIC-R+M (for animals)	Tokyo Chemical Industry	Cat#T3741

**Software and algorithms**		

Imaris	https://imaris.oxinst.com/	N/A
ImageJ	Nat. Methods. 2012; 9:676-682	https://imagej.net/software/fiji/

**Other**		

Round coverslips (15 mm),	Marienfeld,	AP-0111550
60-mm Plastic dishes,	Alpha Plus	No.16021;
Scalpel,	Aesculap Scalpel Handle	No.4 BB083R.
Cimarec Digital Stirring Hotplate,	Thermo Fisher Scientific,	SP142020-33Q
60-mm Plastic dishes,	Alpha Plus	No.16021
Hybridization oven,	Bioman	DHO-100 110V
3D printer	Phrozen	N/A
Resin	Phrozen	TR250LV
silicone sealant	Loctite	N/A
UV lamp	Analytik Jena US	UVP BLAK-RAY B100AP

### Experimental model and study participant details

#### Animals

Thy1-YFP-H transgenic mice (Jackson strain No. 003782),^50^ Fos2A-iCreER knockin mice (Trap2; Jackson strain No. 030323),^53^^,^^54^ and Ai75 reporter mice (Jackson strain No. 025106) were obtained from The Jackson Laboratory. Ai75 mice express nuclear-localized tdTomato upon Cre-mediated recombination. Trap2 mice crossed with Ai75 allow activity-dependent labeling of neurons by expressing nuclear tdTomato after tamoxifen-induced activation of the c-Fos promoter. No sex-based differences were specifically evaluated in this study. Therefore, the potential influence of sex on the reported results cannot be excluded and is acknowledged as a limitation.

All mice were housed in the Institute of Molecular Biology, Academia Sinica under controlled temperature and humidity. Animal procedures were approved by the Institutional Animal Care and Utilization Committee (Protocol Nos. 14-11-759 and 18-10-1234) and conducted in accordance with established guidelines. All mice used in imaging experiments were aged 2 months or older.

The sub-adult bigfin reef squid (*Sepiteuthis lessoniana*) were caught from the natural marine environment near the northeastern inshore of Taiwan. They were kept in an aerated flow-through and filtered seawater system (with an approximate volume of 40,000 L, 25°C seawater in a reinforced concrete pond) with 14/10 h day and night cycle at the Marine Research Station (MRS) of the Institute of Cellular and Organismic Biology, Academia Sinica. Animals were fed three times per day with appropriately sized live Palaemon shrimps. When the animals grew and became sexually mature, they were transferred to a reinforced concrete reservoir with the artificial bamboo groves for egg-laying. After the squid began to exhibit egg capsule-laying behavior, researchers collected the eggs and transferred them to another fiber-reinforced plastic breeding tank. Eggs were checked with a stereomicroscope (Olympus SZ61, Japan) every 8 h for signs of fertilization and embryonic/larval development and maintain to certain stage for further experiment.

### Method details

#### Custom water-immersion cover lens assembly

The BK7 cover lens was designed to compensate for aberrations arising from the water–air refractive index mismatch at the sample interface. To support the lens and allow stable operation under water immersion, a custom cap was fabricated using a stereolithography 3D printer (Phrozen) with engineering high-temperature resin (TR250LV). After printing, the cap was washed and fully post-cured according to the manufacturer’s protocol. The BK7 cover lens was mounted at the front opening of the cap and initially sealed using clear silicone sealant (Loctite). The remaining gap between the cap and the lens was then filled with the same TR250LV resin and UV-cured using a UV lamp (UVP BLAK-RAY B100AP, Analytik Jena US) to ensure a rigid and watertight assembly.

#### Sample preparation and clearing

##### Mouse brain preparation for PEGASOS clearing

PEGASOS tissue clearing was performed as described previously.^11^ Trap2;Ai75 mice were behaviorally activated (e.g., social interaction) and injected intraperitoneally with 4-hydroxytamoxifen (4-OHT, 50 mg/kg) to induce nuclear tdTomato expression in activated neurons. After 2 weeks, brains were collected for clearing.

Mice were anesthetized and transcardially perfused with cold PBS (containing 10 U/mL heparin) followed by 4% paraformaldehyde (PFA). Extracted brains were post-fixed in PFA at 4°C for 72 h, then washed in PBS (3×10 min). Decolorization was performed using 25% Quadrol at 37°C for 2 days, followed by 3% ammonium hydroxide for 1 day.

Brains were delipidated in serial gradients of tert-butanol (tB; 30%, 50%, 70%) at 37°C for 8 h, 24 h, and 48 h, respectively. Following delipidation, samples were dehydrated in 100% tB for 1–2 days. All steps were carried out on a rocking shaker at 10 rpm. Cleared tissues were immersed in BB-PEG clearing medium until optical transparency was achieved (Figure S2) and were maintained in the same medium during imaging acquisition.

##### Mouse embryo and squid sample preparation for CUBIC-R clearing

CUBIC-R clearing of E15.5 mouse embryos was performed following established protocols.^65^ Pregnant mice were euthanized via pentobarbital overdose, and embryos were harvested at embryonic day 15.5 (E15.5). Embryos were fixed in 4% PFA overnight at 4°C, followed by two 10-minute PBS washes at room temperature.

For delipidation, samples were incubated in CUBIC-L reagent for several days, washed again in PBS, and transferred into CUBIC-R reagent for refractive index matching. Samples were embedded in CUBIC-R–agarose and immersed in CUBRIC-R-M for stabilization during imaging.

Stage 24 and 27 squid embryos were prepared for tissue clearing to minimize light scattering and enhance the contrast of autofluorescence imaging. The embryos were fixed in 4% PFA in PBS at 4°C overnight. After fixation, the samples incubated in CUBIC-L reagent for several days, washed again in PBS, and transferred into CUBIC-R reagent for refractive index matching. Samples were embedded in CUBIC-R–agarose and immersed in CUBRIC-R-M for stabilization during imaging. This preparation allowed detailed visualization of structural features in squid embryos without requiring antibody labeling or exogenous fluorescent dyes.

#### Image acquisition and processing

Samples were mounted onto the imaging stage using either superglue or a micro insect pin, depending on tissue type and geometry. For specimens exceeding the field of view (FOV) of the detection objective, the six bounding coordinates—upper, lower, left, right in XY, and start and end positions in Z—were defined using Micro-Manager 2.0. A position list was automatically generated using the “Create Grid” function, with approximately 15% tile overlap to ensure robust alignment during stitching.

Image acquisition was automated using a custom BeanShell script in Micro-Manager, which read the position list and executed non-stop progressive Z-motion acquisition for each tile. This minimized mechanical delay and enabled efficient volumetric imaging. The camera frame rate was set to 10 Hz, and imaging was synchronized with an external trigger from a function generator, which also produced the galvo scanning waveform to coordinate light sheet motion with camera exposure.

Following acquisition, Z-stacks from each tile were downsampled using 2×2 or 4×4 binning to reduce data size and processing time. The binned image stacks were then stitched using the Grid/Collection Stitching plugin in Fiji.

Post-acquisition processing, including 3D rendering, segmentation, and quantitative analysis, was conducted using Amira, Imaris, or Fiji, depending on the imaging task. These tools enabled detailed visualization of both anatomical and cellular features across whole cleared samples.

### Quantification and statistical analysis

The resolution is estimated by measuring the full width at half maximum (FWHM) of dendritic fibers. The signal-to-background ratio (SBR) was calculated as the mean intensity of the cell body divided by the mean intensity of the background.

## Supplemental information

Document S1. Figures S1–S4 and Table S1

Data S1. Technical hardware design and software automation package for the cBLX system, containing the parts list, lens layout, and hardware control macros
