# Supplementary material for: Scanning Bessel beam microscopy with a protected and corrective objective for solvent-cleared large samples
Source: iScience. 2026 Jun 12;29(7):116358. doi: 10.1016/j.isci.2026.116358 (PMC13276785; doi:10.1016/j.isci.2026.116358)
Supplement: Document S1. Figures S1–S4 and Table S1 [file mmc1.pdf]

iScience, Volume *29*

Supplemental information

Scanning Bessel beam microscopy with a protected and corrective objective for solvent-cleared large samples

Chia-Ming Lee, Po-Yen Lin, Yu-Ting Tseng, Xuejiao Tian, Chiao-Hui Tu, José Jiun-Shian Wu, Hsin Chen, Yi-Fen Cheng, Po-Ting Lin, Tung-Han Hsieh, Tzyy-Nan Huang, Tsan-Ting Hsu, Yijuang Chern, Yi-Ping Hsueh, and Bi-Chang Chen

Supplementary information

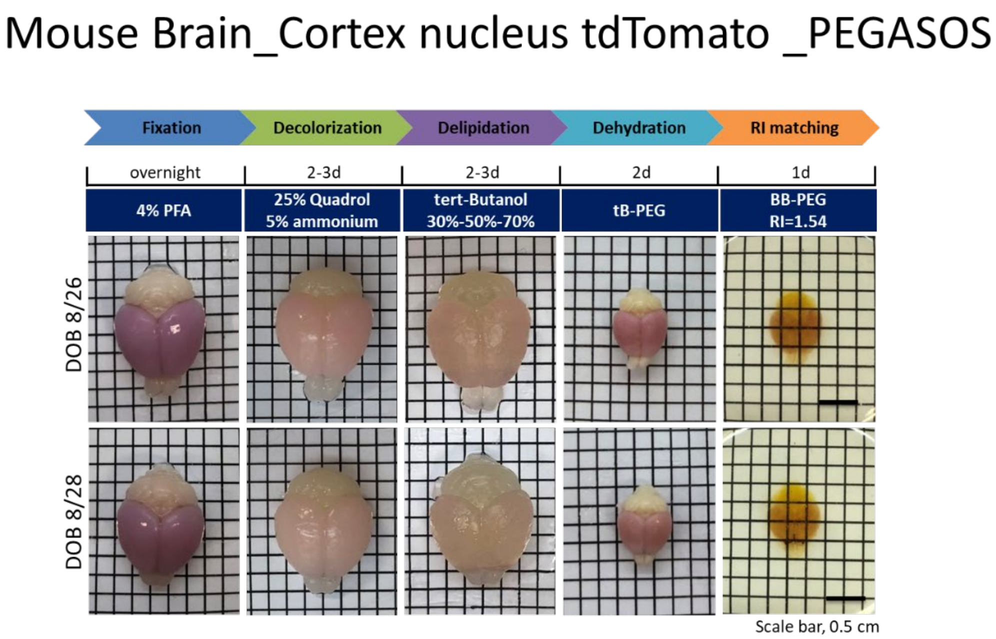

Fig. S1, PEGASOS clearing achieves whole brain transparency and enables 3D imaging with light sheet microscopy.

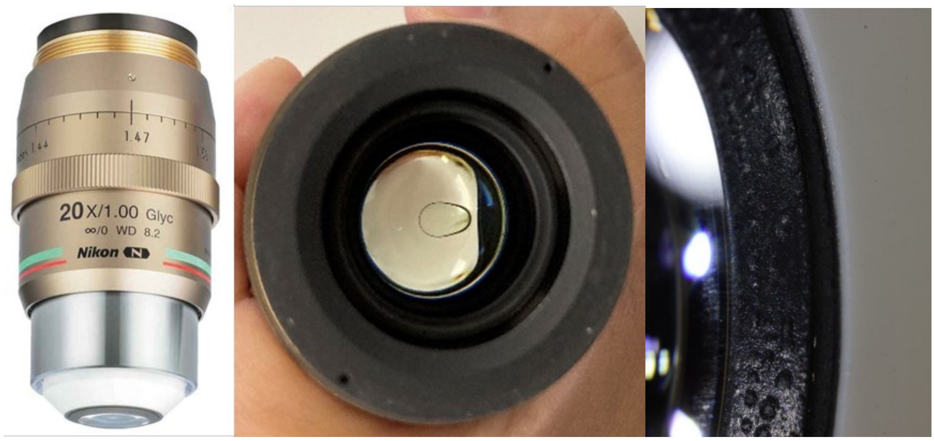

Fig. S2. Photo of the objective lens. Lens coating and sealing damage after immersion of solvent-base clearing medium.

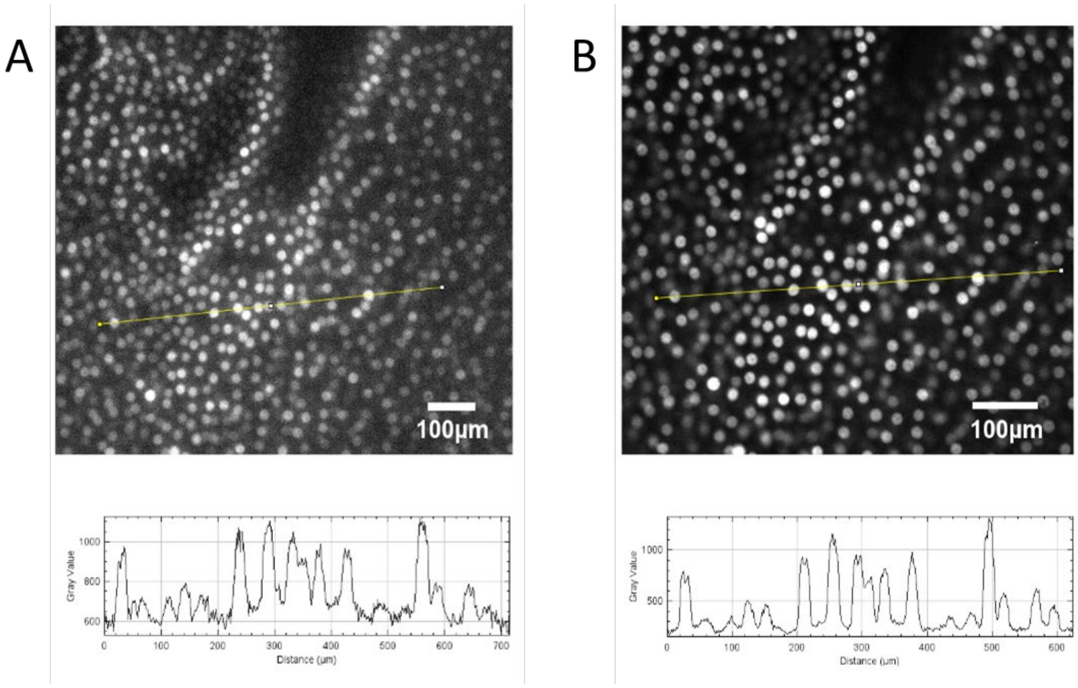

Fig. S3. Light sheet images of expanded zebrafish liver stained with DAPI and acquired by an air objective lens without (A) and with (B) custom lens cap. Image taken with custom lens cap shows superior image contrast.

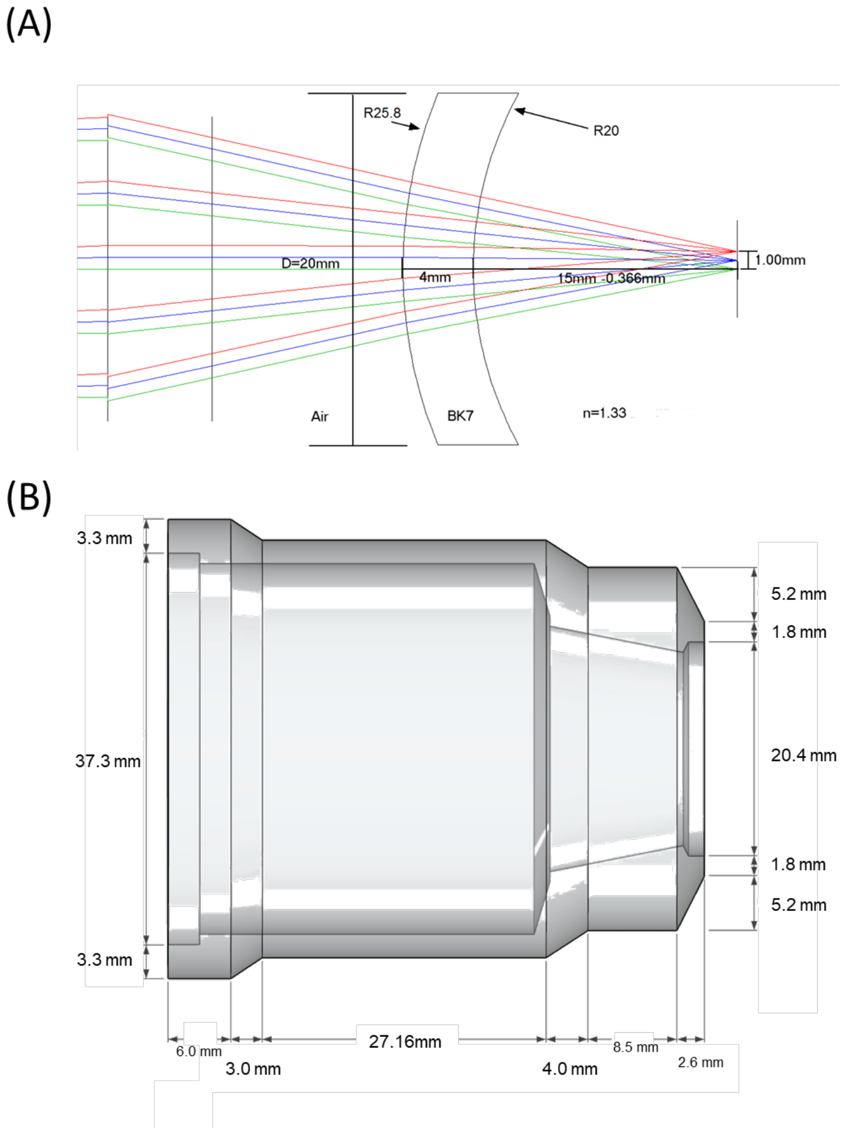

Fig. S4. **Custom cover lens assembly for water-immersion imaging. (A)** Optical design of the BK7 cover lens used to compensate for aberrations caused by the water–air refractive index mismatch at the sample interface. Ray-tracing simulation shows the correction of the optical path when imaging through the water interface. **(B)** Mechanical implementation of the cover lens assembly. A custom cap was fabricated using stereolithography 3D printing with TR250LV high-temperature resin. The BK7 lens was mounted at the front opening of the cap and sealed using silicone, followed by filling the remaining gap with UV-curable TR250LV resin to ensure a stable and watertight structure suitable for water-immersion operation.

Supplementary table 1. Characterization of the system

Objective	Resolution[1]		FOV (μm)	Exposure time (ms)	SBR[2]
	x,y, (μm)	z (μm)			
Nikon 20x	3.4	21.5	880	85	3.77
Olympus 4x	4.5	36	1940	85	3.35
Olympus 4x with cap	3.5	17.75	1462	85	4.16

1. Resolution is estimated via the FWHM of dendritic fibers.
2. The signal-to-background ratio (SBR) was calculated as the mean intensity of the cell body divided by the mean intensity of the background.
