# Supplementary figures and images for: Scanning Bessel beam microscopy with a protected and corrective objective for solvent-cleared large samples
Source: iScience. 2026 Jun 12;29(7):116358. doi: 10.1016/j.isci.2026.116358 (PMC13276785; doi:10.1016/j.isci.2026.116358)

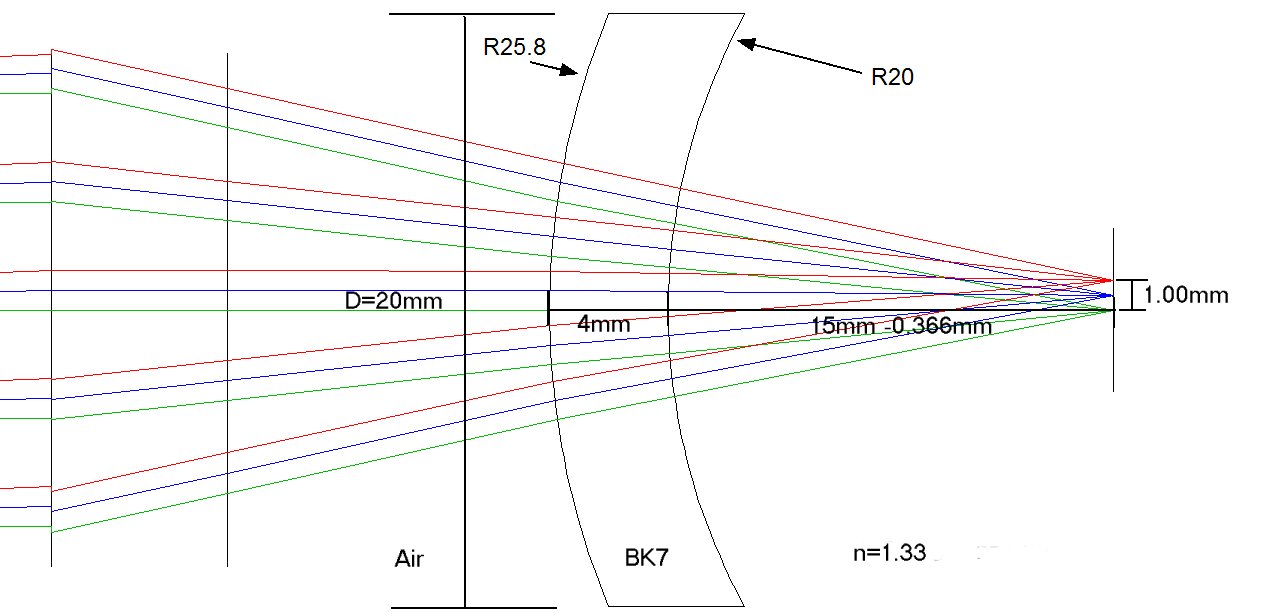

Supplement: Data S1. Technical hardware design and software automation package for the cBLX system, containing the parts list, lens layout, and hardware control macros [file mmc2.zip › BLX_supplemental files/cap_lens.jpg]

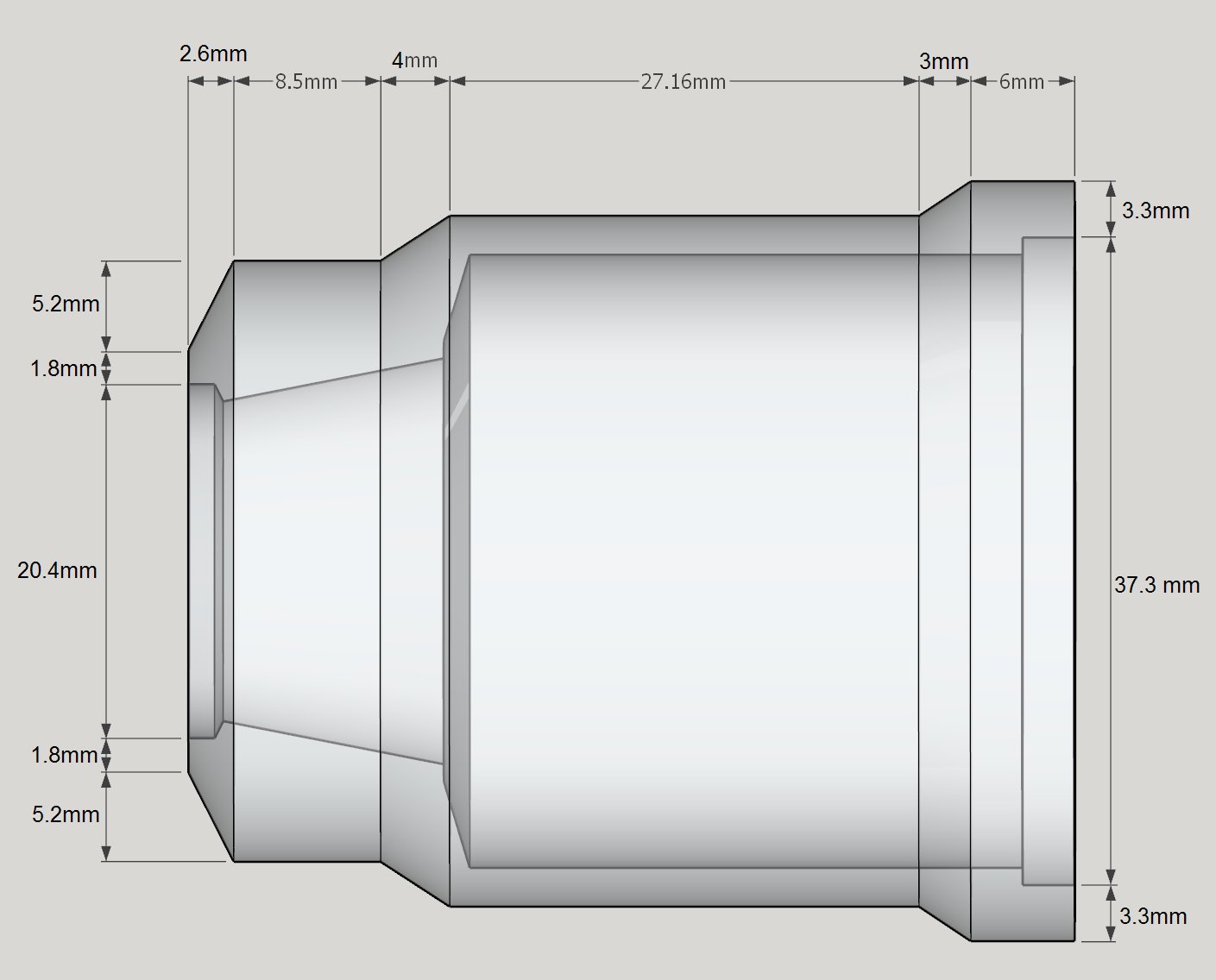

Supplement: Data S1. Technical hardware design and software automation package for the cBLX system, containing the parts list, lens layout, and hardware control macros [file mmc2.zip › BLX_supplemental files/lens_cap_3d_design.jpg]
